# Bio-Organic Fertilizer Promotes Pear Yield by Shaping the Rhizosphere Microbiome Composition and Functions

**DOI:** 10.1128/spectrum.03572-22

**Published:** 2022-12-01

**Authors:** Zhonghua Wang, Tianjie Yang, Xinlan Mei, Ningqi Wang, Xiaogang Li, Qingsong Yang, Caixia Dong, Gaofei Jiang, Jing Lin, Yangchun Xu, Qirong Shen, Alexandre Jousset, Samiran Banerjee

**Affiliations:** a Jiangsu Provincial Key Laboratory for Organic Solid Waste Utilization, Key Laboratory of Plant immunity, Jiangsu Collaborative Innovation Center for Solid Organic Waste Resource Utilization, National Engineering Research Center for Organic-based Fertilizers, Nanjing Agricultural University, Nanjing, China; b Institute of Pomology, Jiangsu Academy of Agricultural Sciences, Jiangsu Key Laboratory for Horticultural Crop Genetic Improvement, Nanjing, China; c Department of Microbiological Sciences, North Dakota State University, Fargo, North Dakota, USA; Nanjing Institute of Geography and Limnology, Chinese Academy of Sciences

**Keywords:** bioorganic fertilizers, pear yield, plant growth-promoting rhizobacteria, rhizosphere microbiome

## Abstract

Bio-organic fertilizers (BOF) containing both organic amendments and beneficial microorganisms have been consistently shown to improve soils fertility and yield. However, the exact mechanisms which link amendments and yields remain disputed, and the complexity of bio-organic fertilizers may work in parallel in several ways. BOF may directly improve yield by replenishing soil nutrients or introducing beneficial microbial genes or indirectly by altering the soil microbiome to enrich native beneficial microorganisms. In this work, we aim to disentangle the relative contributions of direct and indirect effects on pear yield. We treated pear trees with either chemical fertilizer or organic fertilizer with/without the plant-beneficial bacterium Bacillus velezensis SQR9. We then assessed, in detail, soil physicochemical and biological properties (metagenome sequencing) as well as pear yield. We then evaluated the relative importance of direct and indirect effects of soil amendments on pear yield. Both organic treatments increased plant yield by up to 20%, with the addition of bacteria tripling the increase driven by organic fertilizer alone. This increase could be linked to alterations in soil physicochemical properties, bacterial community function, and metabolism. Supplementation of organic fertilizer SQR9 increased rhizosphere microbiome richness and functional diversity. Fertilizer-sensitive microbes and functions responded as whole guilds. Pear yield was most positively associated with the *Mitsuaria*- and *Actinoplanes*-dominated ecological clusters and with gene clusters involved in ion transport and secondary metabolite biosynthesis. Together, these results suggested that bio-organic fertilizers mainly act indirectly on plant yield by creating soil chemical properties which promote a plant-beneficial microbiome.

**IMPORTANCE** Bio-organic fertilization is a widely used, eco-friendly, sustainable approach to increasing plant productivity in the agriculture and fruit industries. However, it remains unclear whether the promotion of fruit productivity is related to specific changes in microbial inoculants, the resident microbiome, and/or the physicochemical properties of rhizosphere soils. We found that bio-organic fertilizers alter soil chemical properties, thus manipulating specific microbial taxa and functions within the rhizosphere microbiome of pear plants to promote yield. Our work unveils the ecological mechanisms which underlie the beneficial impacts of bio-organic fertilizers on yield promotion in fruit orchards, which may help in the design of more efficient biofertilizers to promote sustainable fruit production.

## INTRODUCTION

The rhizosphere microbiome is indisputably important for plant growth and health (1, 2). Microbes in the vicinity of plant roots provide a range of benefits for the host plants, including development (3, 4), nutrition (5, 6), protection against soilborne pathogen infections (7, 8), and tolerance to abiotic stresses (9, 10). Thus, connecting the structure, function and ultimately the effect on plant yield of the rhizosphere microbiome is a crucial step for sustainable crop production in agriculture (11). Previous studies have characterized the microbiome assemblies and functions in rhizosphere soils of model and crop plants, including *Arabidopsis* (12), *Medicago* (13), potato (14), barley (15), maize (16), rice (17), and soybean (18) as well as few non-crop fruit tree species, including grapevine (19), citrus (20), and pear (21, 22). However, the majority of rhizosphere microbiomes in plants have been determined by amplicon-based sequencing approaches (23). Metagenomics can provide taxonomic, genomic, and functional profiles of microbiomes for building better connections between potential microbiomes, functional genes, and yield (24). These advanced metagenomic studies have been well established for human (25), oceanic (26), soil (27), and rhizosphere microbiomes in model and crop plants (28), but rarely for fruit trees (29), particularly pear (21). Thus, metagenomic analyses would enable a deeper understanding of the impacts of the functional microbiome, including community composition and functions, on fruit production.

Pear (*Pyrus* spp.) is one of the most important fruit species worldwide and is particularly important in China (30). The planting area and annual yield of Chinese pear form the largest global industry, accounting for 80.5% of pear production worldwide (31). Intensive pear production is generally considered a system with high fertilizer input, especially excessive and long-term application of chemical fertilizers for high economic returns in China (32). Such excessive chemical fertilization has caused serious environmental degradation, such as reactive nitrogen loss, soil acidification, and global warming (33–35), which in turn has limited pear production (32). Therefore, organic and bio-organic fertilizers are recognized as eco-friendly and effective resources to maintain soil fertility and promote crop yield (36). Furthermore, bio-organic fertilizers containing plant growth-promoting rhizobacteria (PGPRs) and organic substrates that mobilize soil nutrients and improve plant health result in enhanced crop production (37). Many commercial bio-organic fertilizers have been produced that integrate PGPRs with mature compost for yield promotion and disease prevention (38). Among the numerous PGPRs, bacteria within the genus *Bacillus* are ubiquitous in both soil (39) and rhizosphere ecosystems (40) and are widely recognized for their plant growth-promoting potential (41). Many *Bacillus* strains within the rhizosphere exert beneficial effects on plant growth, resistance stimulation, and nutrient uptake by mineralizing organic matter (41). However, there are few reports concerning the effects of bio-organic fertilizers with *Bacillus* strains on the productivity, nutrient availability, and rhizosphere microbiome of pear in the open-field environment. Deciphering the ecological mechanisms of bio-organic fertilizer on yield promotion might help in the design of more efficient biofertilizers to promote sustainability in fruit production.

Bacillus velezensis SQR9 (previously *B. amyloliquefaciens* or B. subtilis SQR9) was isolated from the rhizosphere soil of healthy cucumber plants (42) and it displays significant growth promotion and plant protection abilities on vegetables and crops (43–50). This study hypothesized that the application of bio-organic fertilizers with *B. velezensis* SQR9 would promote pear yield due to compositional and functional changes in the rhizosphere microbiome. To test this hypothesis, a 3-year field experiment in a pear orchard under chemical, organic, and bio-organic (organic fertilizer amended with SQR9) fertilizer regimes was conducted to analyze the responses of the rhizosphere microbiome via whole-genome sequencing-based metagenomics. The objectives were to (i) evaluate the impacts of different fertilization schemes on pear yields, (ii) determine changes in rhizosphere abiotic physicochemical and biotic microbiome properties due to fertilizer treatments, and (iii) determine relationships between fertilizer treatments, soil physicochemical properties, and the microbiome rhizosphere and their impacts on pear yields.

## RESULTS

### Fertilization scheme alters the pear yield and rhizosphere physiochemistry properties.

**FIG 1:**
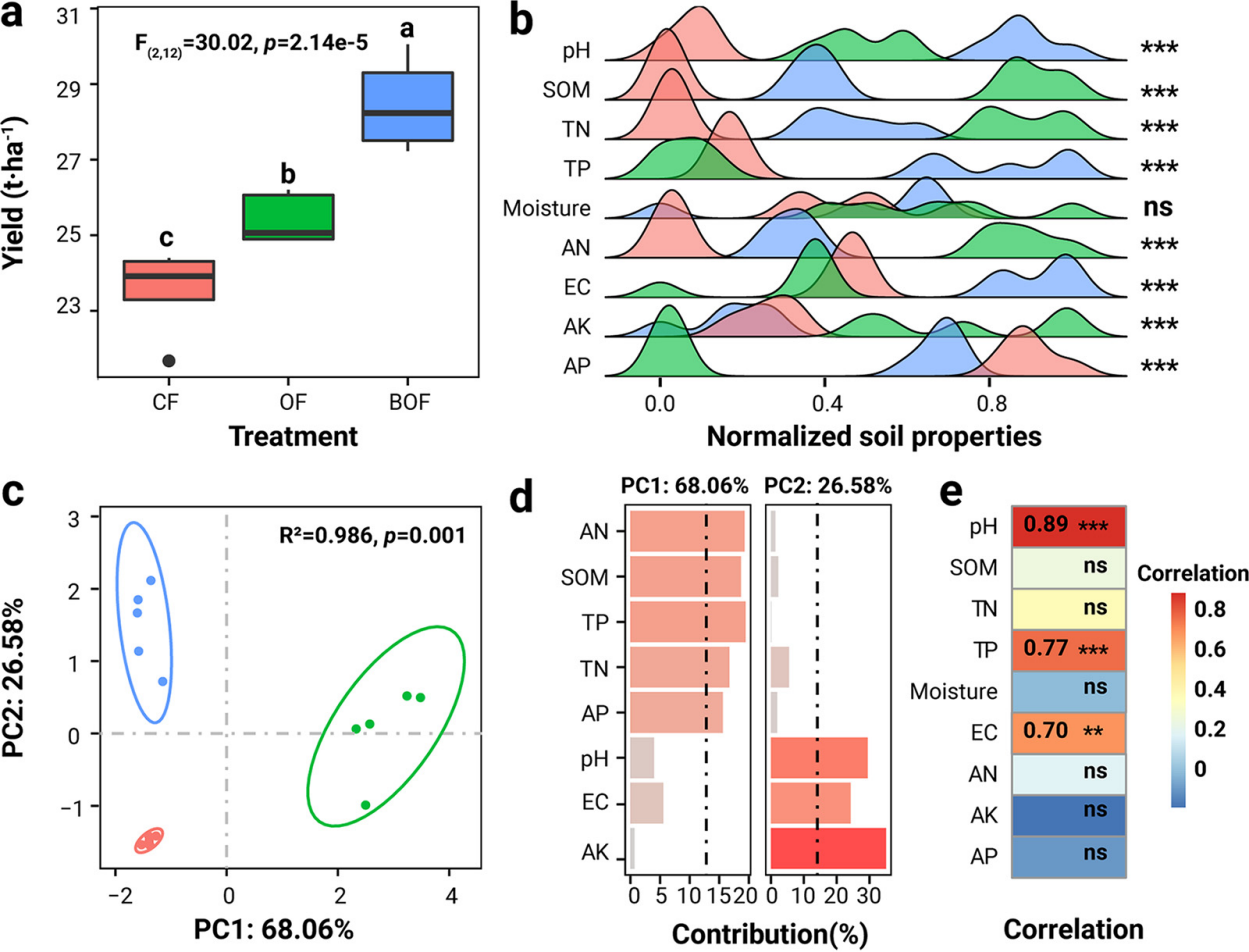
Differences in pear yield and soil abiotic properties between fertilization schemes. (a) Means of pear yield (*n* = 5) in three different fertilization treatments. Different small letters above box plots denote significant differences between treatment groups (*P* < 0.05). (b) Variations in normalized soil abiotic factors. (c) Principal-component analysis (PCA) reveals distinct soil abiotic properties among treatment. “ns” denotes non-significant differences (*P > *0.05) and asterisks (***) denote significant differences of *P* < 0.001. (d) Contribution of each soil abiotic property to first two PC axes. (e) Correlations between yield and soil abiotic properties. ***, *P* < 0.001; **, *P* < 0.01; ns, *P > *0.05. CF, chemical fertilizaer; OF, organic fertilizer; BOF, bio-organic fertilizer.

Over 3 years of fertilization, pear yield was significantly affected by different fertilizers (analysis of variance [ANOVA]: *F*_2,12_ = 30.02, *P < *0.001; Fig. 1a). Pear yield was 1.12 times higher in bio-organic fertilizer with *B. velezensis* SQR9 (BOF)-treated plants compared to organic fertilizer (OF)-treated plants (*P < *0.05), and 1.21 times higher than chemical fertilizer (CF)-treated plants (*P < *0.05); pear yield in OF plants was 1.08 times higher than that in CF plants (honestly significant difference [HSD] test: *P < *0.05; Fig. 1a). We then compared soil abiotic physicochemical factors among different fertilization schemes and assessed their associations with pear yields. Physicochemical properties, including pH, soil organic matter (SOM), available phosphorus (AP), total nitrogen (TN), total phosphorus (TP), available nitrogen (AN), electronic conductivity (EC), available potassium (AK) and available phosphorus (AP), differed considerably between fertilization schemes (*P < *0.05, Fig. 1b and Fig. S1). In particular, pH, SOM, TN, and AN in the rhizosphere soil of CF plants were significantly lower than in that of and BOF plants, while more AP in CF plants was found relative to that in OF and BOF plants (Fig. 1b and Fig. S1). Principal-component analysis (PCA) of physicochemical properties showed that rhizosphere samples clearly formed three different clusters based on fertilization scheme (permutational multivariate ANOVA [PERMANOVA]: *R^2^* = 0.968, *P = *0.001; Fig. 1c). The first two principal components (PCs) accounted for more than 94% of the variance in rhizosphere soil chemical properties (Fig. 1c). PC1 was responsible for 68.06% of soil physicochemical variance due to variations in AN (19.28%), SOM (18.67%), TN (16.72%), AP (19.45%), and AK (15.61%), while PC2 accounted for 26.58% of soil physicochemical variance due to differences in TP (29.46%), pH (35.01 %), and EC (24.27%) as is shown in Fig. 1d. Correlation analysis revealed that pH (*R *=* *0.89, *P < *0.001), TP (*R *=* *0.77, *P < *0.001), and EC (*R *=* *0.70, *P* = 0.0038) were positively associated with pear yield (Fig. 1e). Together, these results suggested that fertilization regimes can improve pear yield through the alteration of soil physicochemical properties.

### Differences in rhizo-microbiome are associated with edaphic variations among fertilization types.

**FIG 2:**
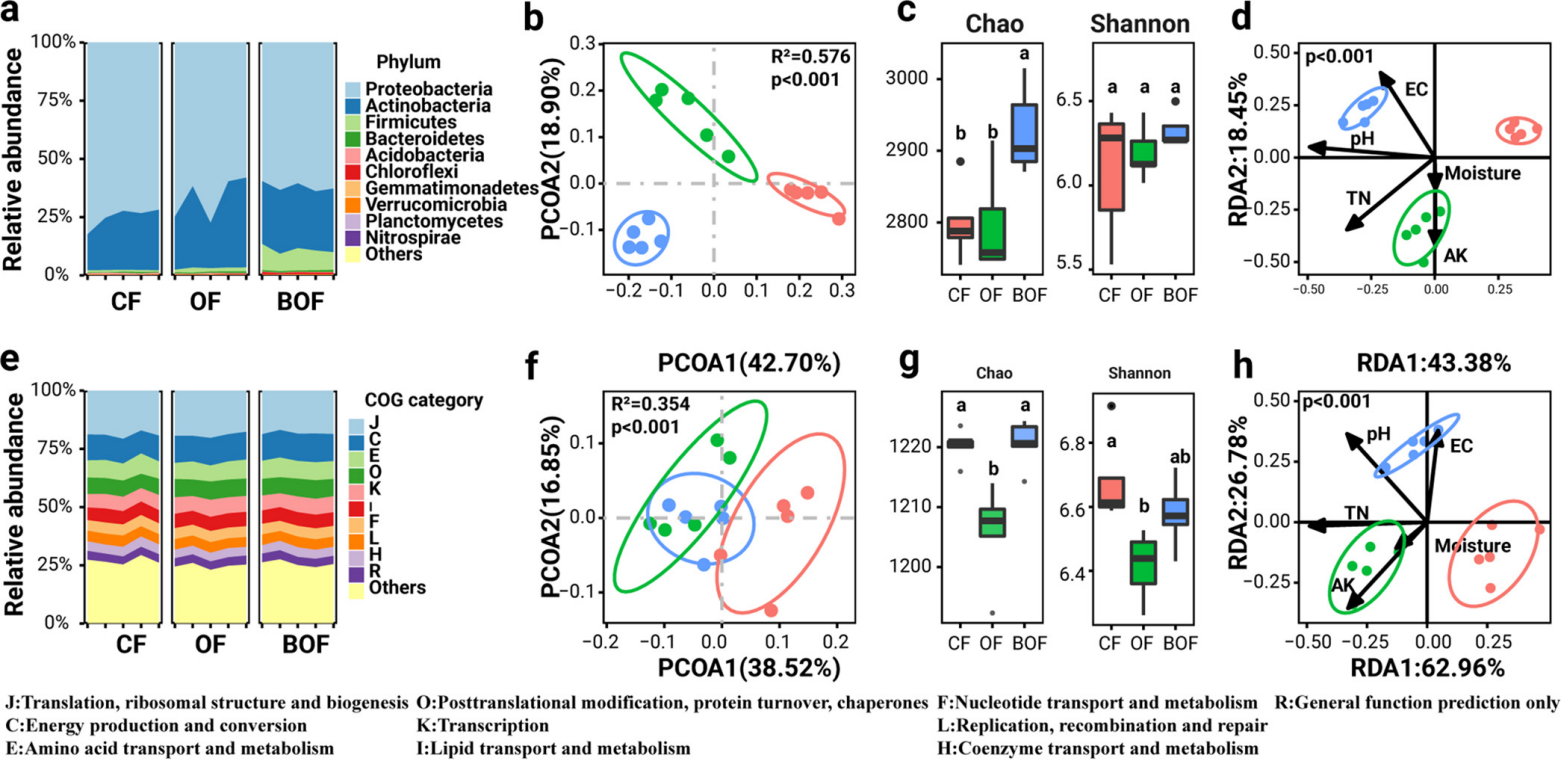
Differences in rhizosphere microbiome across three fertilization schemes. (a) Taxonomic features of top 10 abundant phyla in rhizosphere bacterial communities under different treatments. “Others” denotes rare phyla. (b) Bacterial community diversity (Chao1 richness index and phylogenetic diversity index) under different treatments. (c) Principal-coordinates analysis (PCoA) based on Bray-Curtis distance reveals distinct soil bacterial community clusters relative to treatment groups. (d) Redundancy analysis (RDA) reveals relative importance of soil abiotic properties in taxonomic variations. (e) Top 10 abundant cluster of orthologous genes (COG) categories of bacterial functional profiles. “Others” denotes rare genes. (f) Functional diversity (Chao1 richness index and phylogenetic diversity index) under different treatments. (g) PCoA analysis based on Bray-Curtis distance reveals distinct functional composition of bacterial community relative to treatment groups. (h) RDA analysis reveals relative importance of soil abiotic properties in functional variations. Explanatory power (bar) and significance (**, *P* < 0.01) of individual properties are shown on the right.

Metagenomic profiling revealed considerable differences in the taxonomic composition, diversity, and functional profiles of bacterial communities in pear rhizosphere soils across different fertilization schemes. *Proteobacteria*, *Actinobacteria*, and *Firmicutes* were the dominant bacterial phyla, accounting for an average relative abundance of 98.32% in bacterial communities in the pear rhizosphere (Fig. 2a). Among 3,887 annotated rhizosphere bacteria, *Bacillus* spp. (*F*_2,13_ = 113.7, *P* < 0.001), *Mycolicibacterium* spp. (*F*_2,13_ = 23.74, *P* < 0.001), *Sphingobium* spp. (*F*_2,13_ = 57.57, *P* < 0.001) were significantly different among the three treatments of pear plant rhizosphere soils (Fig. S2a) and positively associated with pear yields (Fig. S2b and Table S2). Compared to those of CF and OF plants, the rhizosphere soils of BOF plants were enriched with *Proteobacteria* but depleted in *Firmicutes* (Fig. 2a). Furthermore, principal-coordinate analysis (PCoA) revealed that fertilization schemes explained 57.6% of total variations in bacterial community compositions (PERMANOVA: *R^2^* = 0.576, *P* < 0.001; Fig. 2b). Compared to the difference of rhizosphere microbiome composition between OF and CF (*R*^2^ = 0.45, *P* = 0.024), BOF differed more from CF treatment in rhizo-bacterial community composition (*R*^2^ = 0.60, *P* = 0.027). The Chao1 richness index (*F*_2,13_ = 7.173, *P* = 0.009) of bacterial communities were significantly different among fertilization schemes (ANOVA; Fig. 2c). Redundancy analysis revealed that soil physicochemical properties were responsible for more than 60% of rhizosphere microbiome taxonomic variation (*R^2^* = 0.689, *P* < 0.001; Fig. 2d), mainly contributed by soil pH, TN, and EC.

Functional annotation discovered a total of 2,583 annotated clusters of orthologous genes (COGs) and revealed that the most abundant functions included the categories J (translation, ribosomal structure, and biogenesis), C (energy production and conversion), E (amino acid transport and metabolism), O (posttranslational modification, protein turnover, chaperones), K (transcription), I (lipid transport and metabolism), F (nucleotide transport and metabolism), L (replication, recombination and repair), and H (coenzyme transport and metabolism), accounting for 74.21% of microbiome functional profiles in the pear rhizosphere soils (Fig. 2e). However, the gene abundance of functional categories was not different between fertilization schemes or associated with yields. A total of 35 differential COGs were enriched in BOF and depleted in CF and OF (Fig. S3a) and positively correlated with yield (Fig. S3b and Table S3). Specifically, the abundance of COG1454 (alcohol dehydrogenase), COG0686 (alanine dehydrogenase), COG4663 (extracellular solute-binding protein, family 7), and COG0474 (P-type ATPase) were positively correlated with yield (*R^2^* > 0.6, *P* < 0.001; Table S3). Furthermore, PCoA revealed that COG composition significantly differed between fertilization schemes, accounting for 61.6% of functional variations (PERMANOVA: *R^2^* = 0.354, *P* < 0.001; Fig. 2f). However, organic fertilizer (*R^2^* = 0.35, *P* = 0.036) and bio-organic fertilizer (*R^2^* = 0.33, *P* = 0.027) were almost equally different from chemical fertilizer in their COG functional profiles. The Chao1 richness indices (*F*_2,13_ = 18.89, *P* < 0.001) and Shannon indices (*F*_2,13_ = 6.54, *P* = 0.0119) of functional profiles were significantly different among fertilization schemes (ANOVA; Fig. 2g). Redundancy analysis (RDA) revealed significant effects of soil physicochemical and microbiome properties on functional composition (Monte Carlo test: *R^2^* = 0.716, *P* < 0.001; Fig. 2h). The first two RDAs were responsible for 88% of functional variations and were tightly associated by TN, AK, EC, and Chao1 index (*P* < 0.05).

### Fertilization schemes shape the bacterial co-occurrence patterns in rhizosphere.

**FIG 3:**
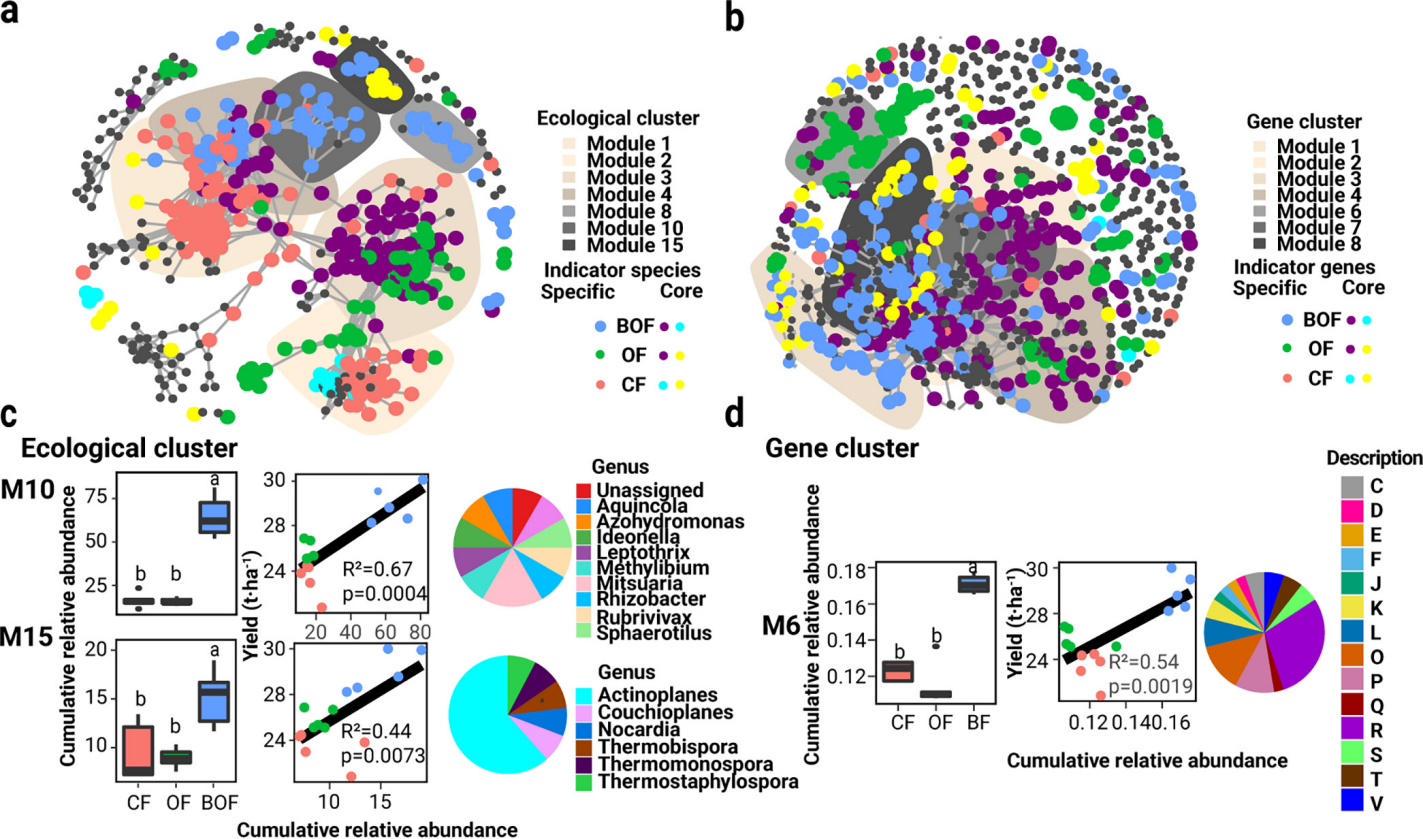
Co-occurrence patterns of indicator species and functions. (a) Co-occurrence networks visualizing significant correlations (*R *>* *0.9, *P* < 0.05) between bacteria and gene indicators in bacterial communities and functional profiles. Colors indicate the different fertilization schemes. Shaded areas (modules) represent ecological (nodes > 10) and functional modules (nodes > 20) of the co-occurrence network indicators as in Tables S5 and S7. (b) Cumulative abundance of each ecological and gene cluster module. Cumulative abundance with significant differences between samples from different fertilization schemes are shown here, other modules are listed in Tables S8 and S9. BOF, blue; OF, green; CF, red. (c) Qualitative taxonomic composition of cropping sensitive modules is reported as proportional operational taxonomic unit numbers per genus (bacteria) and significant correlations (*P* < 0.05) between pear yield and cumulative relative abundance of each ecological cluster modules. (d) Qualitative gene composition of cropping sensitive modules is reported as COG (functions) and significant correlations (*P* < 0.05) between pear yield and the cumulative relative abundance of each gene cluster module.

The study constructed meta-networks of three different fertilization schemes to explore the distribution patterns of indicator species and genes in taxonomic and functional networks (Fig. 3). In a total of 512 vertices of the bacterial co-occurrence network (Table S4), 364 were indicator species vertices, of which 140 were indicator species of CF, 177 were indicator species of, and 166 were indicator species of BOF (Fig. 3a, Table S4). In a total of 864 vertices of the functional gene co-occurrence network (Table S5), 471 were indicator gene vertices, of which 66 were indicator genes of CF, 311 were indicator genes of, and 288 were indicator genes of BOF (Fig. 3b, Table S6). We found that the abundance patterns of bacterial taxa and genes associations also responded to fertilization scheme (Fig. 3). In particular, 7 out of 33 ecological modules contained more than 10 indicator species among the bacterial co-occurrence network which specifically responded to fertilization types (Table S4). Within the functional gene co-occurrence network, 11 out of 161 functional modules contained more than 10 indicator genes (Table S6).

In particular, the effect of fertilization schemes in the bacterial communities was apparent with discrete modules (M1 and M2) in the taxonomic network, containing indicator species specific to CF fertilization (Fig. 3a). M3 and M15 contained indicator species specific to OF fertilization, while M4, M8, M10, and M15 were mainly occupied by indicator species (mainly *Actinobacteria* and *Proteobacteria*) specific to BOF fertilization (Fig. 3a, Table S5). M1, M2, M3, M4, and M8 are main parts of the network, and M1 and M2, dominated by indicator species of CF and OF, are separated from M8, which was dominated by specific indicator species of BOF. M8, dominated by specific indicator species of BOF, and M15, dominated by indicator species of BOF and OF, are both individual clusters. For the functional network, M1 was enriched with indicator genes specific to BOF fertilization while M6 and M7 mainly contained indicator genes specific to OF fertilization (Fig. 3d). M2, M3, and M4 contained indicator genes of BOF and OF fertilization related to metabolism, including energy production and conversion, carbohydrate transport and metabolism, and amino acid transport and metabolism (indicated by C, G, and E, respectively, in Fig. 3d). M1, M2, M3, M4, M7, and M8 are in the main part of the network, while M6 is an individual gene cluster. All the fertilization-responsive modules in ecological and functional networks were comprised of broad clusters of bacteria and genes, revealing that the different fertilization schemes shaped a considerable number of bacterial lineages and functions.

The types of sensitivity of these module members to the specific fertilization schemes (Fig. 3b) and their distribution in the network partially reflected the drivers of bacterial community dissimilarity seen in the PCoA ordinations (Fig. 2c). In detail, the cumulative relative abundance of ecological M10 (dominated by *Mitsuaria*, *F*_2,13_ = 68.58, *P* < 0.001), M15 (dominated by *Actinoplanes*, *F*_2,13_ = 9.269, *P* < 0.001; Fig. S4a), and gene cluster M6 (mainly metabolism genes; inorganic ion transport and metabolism genes [P], *F*_2,13_ = 68.19, *P* < 0.001; Fig. S4b) varied among fertilization schemes (ANOVA, Fig. 3c and d). Within these modules, the bacterial species and functional genes of the BOF treatment group displayed higher abundances (*P* < 0.05) than those of the OF and CF groups, while the latter two groups were not distinguishable from each other (HSD test: *P* > 0.05; Fig. 3c and d). Correlation analysis revealed that the cumulative relative abundance of ecological M10 (*R *=* *0.67, *P* < 0.001), M15 (*R *=* *0.44, *P* = 0.007; Fig. S4a and Table S8), and gene cluster M6 (*R *=* *0.54, *P* = 0.001) were positively associated with pear yield (Fig. 3c and d), while gene clusters M3 and M8 were negatively linked with pear yield (Fig. S4b and Table S9). Together, these results suggested that fertilization schemes can shape co-occurrence patterns in both the taxonomic and functional networks and that BOF fertilization might improve pear yield by enriching the relative abundances of specific ecological and functional modules.

### Overall effect of fertilization schemes on pear yield.

**FIG 4:**
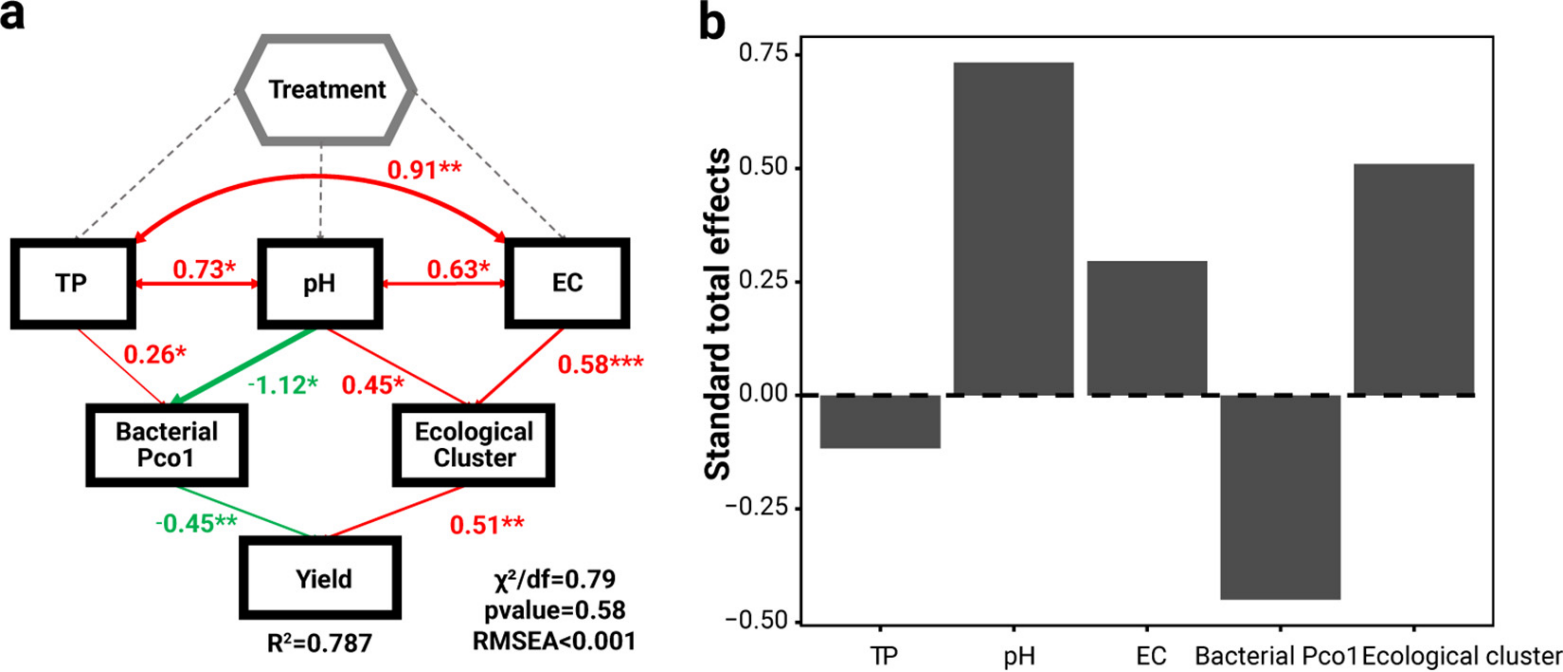
Structure equation models (SEMs) of direct and indirect effects of soil abiotic and biotic properties on pear yield under different fertilization schemes. (a) Bacterial community structure denotes bacterial dissimilarity, referred to as the first coordinate of PCoA analysis. Ecological cluster represents relative abundance of ecological cluster modules 10 and 15, which have significant associations with pear yield. *, *P* < 0.05; **, *P* < 0.01; ***, *P* < 0.001. (b) Standard total effects (STE) of soil abiotic factors, bacterial structure and ecological cluster on pear yield in SEM.

Although considerable differences were observed in pear yield and its relevance to rhizosphere soil abiotic and biotic properties under different fertilization scenarios, there was still a lack of deeper knowledge of overall fertilization effects on pear yield. We used structural equation model (SEM) analysis to explore how fertilization schemes shape pear yield via direct and indirect associations between soil physicochemical and bacterial properties (see details in Methods). The fine SEM model had a reasonable fit, explaining 78% of the variation in the pear yields (χ^2^/df = 0.79, *P* = 0.58; Fig. 4). Significantly positive interactions were found between TP, pH, and EC in soil physicochemical properties (*r *>* *0.63, *P* < 0.05). Among these, soil pH and TP had negative (*r* = −1.12, *P* < 0.05) and positive (*r *=* *0.26, *P* < 0.05) effects on the bacterial structure (dissimilarity among samples), respectively, while pH (*r *=* *0.45, *P* < 0.05) and EC (*r *=* *0.58, *P* < 0.001) positively affected the cumulative relative abundance of ecological clusters (bacterial co-occurrence network modules 10 and 15). Bacterial structure and ecological cluster had negative (*r* = −0.45, *P* < 0.01) and positive (*r *=* *0.51, *P* < 0.05) effects on pear yield, respectively. Among the SEM model parameters, pH, EC, and ecological cluster had strong positive effects on pear yields while bacterial structure had a strong negative effect (Fig. 4b). In conclusion, fertilization schemes can aid in pear production through their effects on soil physicochemical and microbial properties.

## DISCUSSION

This work examined the impact of bio-organic fertilizer on yield promotion in a pear orchard and explored its ecological mechanisms. This study imposed treatments with basic fertilizer and organic fertilizer either lacking or containing (bio-organic fertilizer) an inoculation of *B. velezensis* SQR9, a well-studied plant growth-promoting rhizobacterium (51). Our objective was to disentangle the relative contributions of soil properties, microbiome structure, and functional composition to pear yield under different fertilization schemes. This study found that bio-organic fertilizer (organic fertilizer inoculated with *B. velezensis* SQR9) increased pear yields. The total effect of bio-organic fertilizer was comprised of direct effects on soil nutrient promotion by the addition of organic substrate and indirect effects on resident microbial community shifts in the rhizosphere soils of pear trees, with a particularly important roles of specific bacterial ecological modules and functional gene clusters simulated by the *B. velezensis* inoculant.

### Relative importance of different components of bio-organic fertilizer.

The results indicate that the combination of PGPR agent *B. velezensis* SQR9 and organic fertilizer as bio-organic fertilizers can be effective in promoting pear yield. These findings are in line with previous studies which showed that organic matter amendments alone can increase crop production by improving soil nutrients (52, 53). In addition, the addition of beneficial microorganisms to organic fertilizer can effectively promote nutrient uptake and plant growth and thereby improve yield (54, 55). Although *B. velezensis* SQR9 and indigenous *Bacillus* have not been separated in our experiments, B. subtilis, the original taxonomy of the SQR9 inoculant (42) was only identified in the rhizosphere soil of the bio-organic fertilizer treatment (Fig. S5a). These data supported the assumption that the relative abundance of the metagenome-annotated B. subtilis can indicate *B. velezensis* SQR9 colonization of the pear rhizosphere. Furthermore, a high relative abundance of the SQR9 inoculant was found to promote productivity in pear (Fig. S5a). The yield promotion of the SQR9 inoculant has been also observed in crop and vegetable plants due to its plant-growth-promoting capacities by inducing root exudates (56), activating rhizosphere nutrients (48), and emitting volatile components and phytohormone indole-3-acetic acid (57).

Additionally, the rhizosphere soils treated with bio-organic fertilizer were also enriched in the bacterial genera *Mycolicibacterium* (58), *Novosphingobium* (59), and *Sphingobium* (60, 61), which are associated with soil nutrient transformation (62). It has been reported that the addition of beneficial *Bacillus* spp. bacteria increased the relative abundance of other resident beneficial taxa to enhance plant health (63). This demonstrates the stimulation effect of bio-organic fertilizer on increasing the relative abundance of other beneficial bacteria and fungi, including *Bacillus* spp. in our study. These results suggest that the inputs of PGPR agents are involved in promoting plant productivity via direct effects on soil nutrients and indirect effects on rhizosphere microbiome alteration, in line with previous work (52, 53).

### Differential responses of soil abiotic and biotic properties.

Our study found that fertilizer inputs induced specific alterations in rhizosphere soil properties, including soil physicochemical properties, microbiome composition, and functional gene content (Fig. 1 and Fig. 2). The differences observed are similar to those of previous findings tracking responses to different fertilization management regimes (63–65). Our findings on soil physicochemical properties showed that pH, soil organic matter, total phosphorus, and EC were increased in the BOF treatment group compared to that in the chemical fertilizer treatment group. This is in accordance with other studies that bio-organic fertilizer promotes pH and nutrients, especially soil organic matter, in soil (66). As in previous studies (63, 67), bacterial community composition appeared to be more affected by bio-organic fertilizer treatment than bacterial diversity in response to fertilization-induced changes in soil physicochemical properties (Fig. 2). Furthermore, microbiome alterations in response to fertilizer regimes were good predictors of plant yield (Fig. 4a), suggesting that the positive impacts of organic and bio-organic fertilizer on yield may be mediated by alterations in microbiome composition (64, 68). Notably, we observed specific functional gene responses to different fertilization regimes using metagenomic functional analysis (Fig. 2e and f). In particular, more functional genes involved in energy production and conversion, carbohydrate transport and metabolism, and coenzyme transport and metabolism were observed in the BOF treatment (Table S7), which were associated with physicochemical and microbiome structural variations in rhizosphere soils (Fig. 2g). Among the fertilizer-induced functional variations, a considerable set of functions (35 out of 217 differential COGs, Fig. S3) were tightly linked to pear yield. These COGs encompassed a range of functions, including growth, stress tolerance, metabolism, and nutrient cycling, and we could further identify specific plant-growth promoting genes such as alcohol dehydrogenase (COG1454) and alanine dehydrogenase (COG0686), as listed in Table S3.

### Differential responses of microbial and functional co-occurrences to fertilization types.

The study identified fertilization-sensitive bacterial taxa and functional genes in rhizosphere microbial communities which served as indicators to explain composition patterns by fertilization management regime. Some *Bacillus*, *Sphingobium*, and *Sphingopyxis* species were identified as indicators of BOF treatment, and their relative abundances were positively correlated with pear yield. This result suggested that the combination of organic fertilizer and *Bacillus* SQR9 may promote pear productivity via altering the resident rhizosphere community, especially some specific taxa (69, 70). Furthermore, considering fertilizing indicators, the study identified a set of ecological and functional modules containing high proportions of bacterial taxa and functional genes which responded similarly to different fertilizer inputs (64, 65). The bacterial taxa were observed to be clustered in distinct ecological modules among the CF, OF, and BOF fertilization treatments. These results suggested that these specific groups of bacteria responded to different fertilization types and grouped together in the rhizosphere bacterial networks to promote fruit productivity. The functional genes exhibited low to medium degrees of co-occurrence in the functional network, revealing that fertilization management moderately affects highly co-occurring bacterial functions (71). This may mean that influential bacterial community taxa with distinctly redundant functions can also be manipulated with bio-organic fertilization in the rhizosphere microbiome (72).

### Positive impact of fertilization on the soil properties as pathway to yield promotion.

This work provides evidence that fertilization enhances soil nutrients, pH, and the relative abundance of specific bacterial taxa and functional genes associated with nutrient cycling and plant growth promotion. The SEM analysis provides further evidence that bio-organic fertilization increased the pear yield by decreasing the distinctiveness of bacterial community composition and increasing the relative abundance of specific bacterial taxa within modules 10 and 15 (Fig. 3). *Mitsuaria* spp. and *Actinoplanes* spp. dominated ecological modules 10 and 15, respectively, the cumulative abundances of which were positively associated with pear yield. Similarly, *Mitsuaria* spp. have been shown to promote the biomass of crops such as rice and maize (73, 74) and enhance drought tolerance in fruit plants like jujube and citrus (75, 76). Besides *Mitsuaria* spp., *Actinoplanes* spp. also exhibit plant growth-promoting activities due to their multifunctional roles, such as soil nutrient cycling/stabilization and antibacterial activity, in agricultural production sustainability (77–79). This could be explained by the positive associations between pear yield and the cumulative abundance of gene cluster M6, which is dominated by the microbiome functions of ion transport/metabolism and secondary metabolite biosynthesis/transport (Fig. 3). This work demonstrates the mechanisms underlying the effects of fertilization on yield promotion and its associated abiotic physicochemical, biotic, and microbiome structural and functional features in rhizosphere soils.

In conclusion, this work unveils the ecological mechanisms by which bio-organic fertilizer stimulates functional microbiome for pear fruit productivity by connecting soil metagenome analysis with yield data. These findings have deepened understanding of the linkage between pear yield and the stimulated ecological clusters dominated by *Mitsuaria* spp. and *Actinoplanes* spp., and the function clusters dominated by ion transport or/and secondary metabolite biosynthesis, under bio-organic fertilization. Furthermore, this work provides referable evidence of functional rhizosphere microbiomes potentially associated with pear orchard yield, which could raise farmer awareness of applying products fermented from agricultural organic wastes, e.g., bio-organic fertilizers, to promote fruit yield and achieve sustainable agriculture.

## MATERIALS AND METHODS

### Experimental design and sampling of the field experiment.

The field experiment was conducted in the pear orchard at Lishui Botany Science Base (N31°36′59″-E119°10′38″) of Jiangsu Academy of Agricultural Sciences in Baima County (Jiangsu, China) from 2017 to 2019. Ten-year-old pear trees (cultivar Chuxialǜ) were used as model plants for this work. Chuxialǜ is a popular early-maturing pear cultivar derived from a cross between the *P. pyrifolia* Xizilü and Cuiguan cultivars and grafted upon the root stock of *P. calleryana* Decne (80). Pear trees (within one orchard) were treated with three different fertilization regimens: chemical fertilizer (CF), organic fertilizer (OF), and bio-organic fertilizer (BOF), with ~33 pear trees per treatment. All treatments were applied with the same nutrient standard of N 0.6 kg·plant^−1^, P_2_O_5_ 0.3 kg·plant^−1^, and K_2_O 0.7 kg·plant^−1^. CF treatment was applied with a mixture of 2 kg·plant^−1^ of compound fertilizer NPK 15-15-15 (Sinochem Holdings Corporation Ltd., Shandong, China), 2.17 kg·plant^−1^ urea (N = 46.4%, Sinochem Holdings Co. Ltd., Jilin, China), and 0.59 kg·plant^−1^ potassium sulfate 0-0-50 (K_2_O = 51.0%, Sdic Xinjiang Luobupo Potash Co. Ltd., Xinjiang, China). OF treatment was applied with 24 kg·plant^−1^ organic fertilizer (organic matter 51.44%, N 2.51%, P_2_O_5_ 1.25%, K_2_O 2.92%) containing the same amount of NPK nutrient as the CF treatment. BOF treatment consisted of the OF treatment mixed with a microbial inoculant of live *B. velezensis* SQR9 at a final concentration of 2 × 10^9^ CFU·g^−1^. Both organic and bio-organic organic fertilizers were provided by Jiangyin Lianye Bioscience & Biotechnology Co. Ltd. Fertilizers were applied by digging two 70 × 50 × 30-cm pits approximately 70 cm away from the trunk and refilling them with a mixture of fertilizer and the excavated soil. Excess pears were removed at young fruiting stage, leaving around 150 fruits per plant. In the middle of October 2019, 5 tree replicates from each fertilization scheme were selected for yield assessment and rhizosphere soil collection through the five-spot-sampling method (81). A random 20 fruits from 2 ordinate directions were collected to calculate the average single fruit weight. Theoretical yield (t·ha^−1^) was estimated from the average single fruit weight multiplied by the number of fruits (~150) and number of plants (~33) per treatment per mu (~667 m^2^; 1 ha = 15 mu). According to a previously described method (48, 82), six rhizosphere soils were collected from six directions apart from the fertilizing sites and pooled as one sample for each tree. In detail, the top 5 cm of soil was removed, and fine roots (up to 1-mm diameter) were collected from a depth of 5 to 15 cm. The fibrous roots were collected with a shovel and then gently shaken to obtain the rhizosphere soils. Approximately 10 g of fresh rhizosphere soil per plant was retained and divided into two sealed 5-mL Eppendorf tubes to determine soil physicochemical properties and rhizosphere microbiomes.

### Determination of soil physicochemical properties.

Abiotic physicochemical properties included pH, soil moisture content (% moisture), electric conductivity (us·cm^−1^), soil organic matter (g·kg^−1^), total nitrogen (mg·kg^−1^), total phosphorus (mg·kg^−1^), available nitrogen (mg·kg^−1^), available phosphorus (mg·kg^−1^) and available potassium (mg·kg^−1^). Soil physicochemical properties were mainly measured as described previously (83) following the Chinese standard for soil property determination (www.chinesestandard.net): soil pH (HJ 962–2018), EC (HJ 802–2016), SOM (NY/T 1121.6–2006), TN (LY/T 1228–2015), TP (GB/T 9837–1988), AN (LY/T 1229–1999). The difference between fresh and air-dried soil sample weights was used as a proxy of soil moisture for each rhizosphere sample. AP and PK were extracted with hydrochloric acid and ammonium fluoride and measured using the molybdenum blue method (84).

### DNA extraction and sequencing.

The total DNA was exacted from ~0.25 g of each rhizosphere soil sample using a PowerSoil DNA Isolation kit (Mobio Laboratories, Carlsbad, CA, USA) following the manufacturer’s protocol. DNA quality and quantity were determined using a NanoDrop 1000 Spectrophotometer (Thermo Fisher Scientific, MA, USA). Metagenomic library preparation and sequencing were performed on an Illumina NovaSeq 6000 S4 platform according to the manufacturer’s protocol at Microeco Co., Ltd. (Shenzhen, Guangdong, China). The metagenomic DNA was sonicated to the 350-bp size range, end-repaired, 3′-adenylated, and amplified using Illumina sequencing. Quality checking was conducted by KneadData software based on Trimmomatic and removal of host DNA was conducted by Bowtie2. The rationality and effectiveness of quality checking were estimated by FastQC. An average of 7 GB raw data and 2.2 × 10^7^ clean reads were generated for each metagenomic (Table S1).

### Metagenomic data processing.

Clean reads were generated by removing adaptor sequences, trimming, and removing low-quality reads of raw reads from metagenome sequencing (reads with *N* bases and a minimum quality threshold of 20). The clean reads were further trimmed using Trimmomatic and trimmed reads shorter than 50 bp were discarded. The trimmed reads mapped to the pear genomes, including *P. bretschneideri*, (30, 85), *P. betuleafolia* (86), and *P. pyrifolia* (87) were identified and removed by Bowtie2 software (88), resulting in a total of 100 GB clean reads. Kraken2 and Bracken were used for annotation and abundance assessment of microbial species, resulting in archaea (93,666), bacteria (17,032,193), phage (459), and plants and fungi (77,964) categories. Bacteria were the dominant categories, accounting for 99% of annotated microbes, and were used for the following analyses. The clean reads were functionally annotated by the HUMAnN2, KEGG (www.genome.jp/kegg), and eggNOG databases.

### Data processing and statistics.

**(i) Comparing the yield and soil properties among fertilization schemes.** All analysis was performed under R version 4.0.2. Analysis of variance and Tukey’s honestly significant difference test were used to determine the statistical significance of yield and physicochemical properties during multiple comparisons using the *agricolae* package. Principal-component analysis based on the Euclidean distance of the range-normalized values for soil physicochemical properties was used to visualize differences between fertilization treatments using the *FactoMineR* package. Three screening criteria were used to select the samples and soil physicochemical factors for PCA analysis: (i) the Kaiser-Meyer-Olkin measure (>0.50), (iii) the Bartlett’s test of sphericity (<0.05), and (iii) communality values of >0.5. The main components were applicable for latent root criterion (eigenvalues > 1.0). Permutational multivariate analysis of variance was used to test statistical differences (*P* < 0.05) in overall soil physicochemical properties between treatments with 999 permutations using the *Adonis* function in the *vegan* package. Association of yield and soil properties was estimated by Pearson’s correlation test using the *Hmisc* package.

**(ii) Comparing rhizosphere microbiome differences among fertilization schemes.** Bacterial community composition and functional profiles of COG were ordinated by principal coordinates analysis using Bray-Curtis distance, and differences in rhizosphere soil samples between fertilization treatments were compared with PERMANOVA test (*P* < 0.05, 999 permutations) using the *Adonis* function in the *vegan* package (89). To further measure the relative differences in bacterial community composition and functional profiles of COG between treatments, we used pairwise multi-level comparison PERMANOVA in the *pairwiseAdonis* package using the Bonferroni method to adjust *P* values (90). ANOVA and HSD tests were used to determine the statistical significance of the Chao1 and Shannon indices, as well as the abundance of bacterial genera between different treatments, using the *agricolae* package. A redundancy analysis was used to explore the relationships between soil physicochemical properties and microbiome composition using the *vegan* package.

**(iii) Identifying fertilization sensitive taxa and functional genes.** We employed a correlation-based approach to identify sensitive taxa and functions responsible for different fertilization schemes by using the *multipatt* function in the *indispecies* package. First, we calculated the point-biserial correlation coefficient (*r*) of an operational taxonomic unit’s positive association to one or a combination of fertilization schemes. The analysis was conducted with 999 permutations and considered significant at *P* < 0.05.

**(iv) Co-occurrence network constructions.** Co-occurrence networks were constructed to understand the associations between bacterial members and functional genes within the microbiome following a previous pipeline (64). Briefly, a pairwise Spearman’s correlation matrix was calculated with the *rcorr* function in *Hmisc* package, and *P* values were adjusted using the *fdrtool* package. Bacterial species and gene pairs with a Spearman’s correlation coefficient absolute *R* value of >0.9 and *P* < 0.05 were subjected to build the network, then the *cluster_fast_greedy* function in *igraph* package was used to calculate ecological and gene cluster modules. Ecological and functional networks and modules were visualized based on the *igraph* object.

**(v) Structural equation model analysis.** Structural equation models were computed using the *lavaan* package to evaluate the causalities between fertilization treatment (chemical, organic, and bio-organic fertilizer), soil physicochemical characteristics, microbiome composition, and pear yield. The soil abiotic physicochemical properties included pH, electrical conductivity, moisture, soil organic matter, total nitrogen, total phosphorus, available nitrogen, available phosphorus, and available potassium, while the soil biotic microbiome properties were comprised of bacterial community structure (the first coordinate of PCoA analysis), diversity (Chao1 richness and Shannon indices), and ecological cluster (cumulative relative abundance of total species within the module clusters). Variance inflation factor (VIF) values above 10 were removed to update a fine-fitted model due to critical multicollinearity issues of the formative indicators using the VIF function of *car* package. The SEM model was statistically analyzed by the *semTools* package and model information was exacted using the *parameterEstimates* function in the *lavaan* package. The overall goodness-of fit for SEMs were evaluated by the following criteria: the proportion of chi-square (*χ^2^*) and degrees of freedom (*χ*^2^/df < 2 and *P* > 0.05), the root-mean-square error of approximation (closed zero), a high goodness-of-fit index (>0.8), and a standardized root mean square residual (<0.08).

### Data availability.

The original contributions presented in the study are included in the article/Supplemental Material. All raw reads of the rhizosphere metagenomes described in the manuscript have been deposited in the National Genomics Data Center of the China National Center for Bioinformation (https://ngdc.cncb.ac.cn) under the accession no. PRJCA007662 with assigned accession id: CRA009038.
