# Supplementary material for: Bio-Organic Fertilizer Promotes Pear Yield by Shaping the Rhizosphere Microbiome Composition and Functions
Source: Microbiol Spectr. 2022 Dec 1;10(6):e03572-22. doi: 10.1128/spectrum.03572-22 (PMC9769518; doi:10.1128/spectrum.03572-22)
Supplement: Supplemental file 1 — Fig. S1 to S5. Download spectrum.03572-22-s0001.pdf, PDF file, 0.6 MB [file spectrum.03572-22-s0001.pdf]

Supplementary information

Bio-organic fertilizer promotes pear yield by shaping the rhizosphere microbiome composition and functions

Zhonghua Wang, Tianjie Yang, Xinlan Mei, Ningqi Wang, Xiaogang Li, Qingsong Yang, Caixia Dong, Gaofei Jiang, Jing Lin, Yangchun Xu, Qirong Shen, Alexandre Jousset, Samiran Banerjee

The supplementary information contains five figures. Figure files provide differences in physicochemical properties and functional microbiomes including microbial compositions and functions, as well as their associations with pear yield.

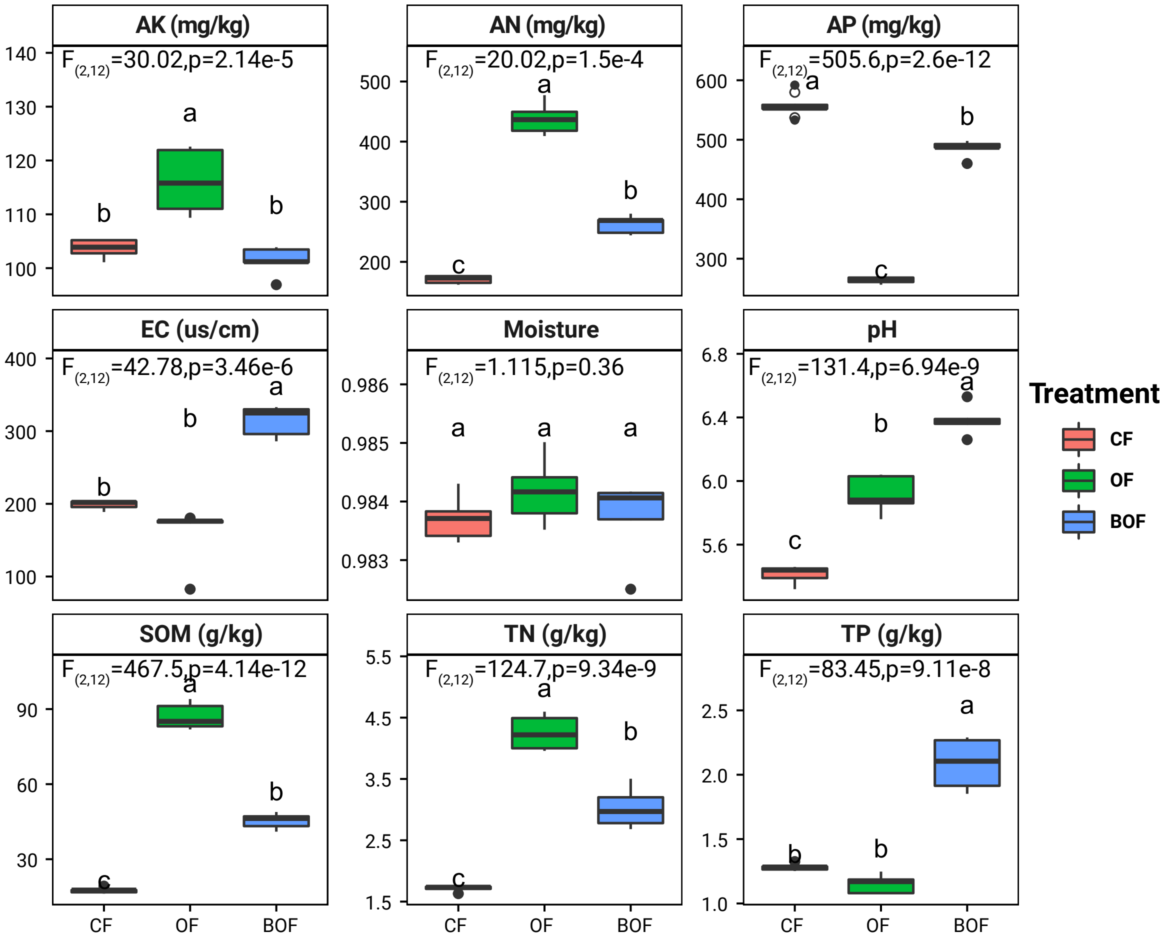

Supplementary Figure 1. Difference in rhizosphere soil physicochemical properties under different fertilization schemes. BOF (blue), OF (green) and CF (red) denote for the treatment of bio-organic fertilizer, organic fertilizer and control fertilizer. Abbreviations and units are the mean of physicochemical properties pH, soil moisture content (Moisture, %), electric conductivity (EC, us·cm^{-1}), soil organic matter (SOM, k·kg^{-1}), total nitrogen (TN, g·kg^{-1}), total phosphorus (TP, mg·kg^{-1}), available nitrogen (AN, mg·kg^{-1}), available phosphorus (AP, mg·kg^{-1}) and available potassium (AK, mg·kg^{-1}).

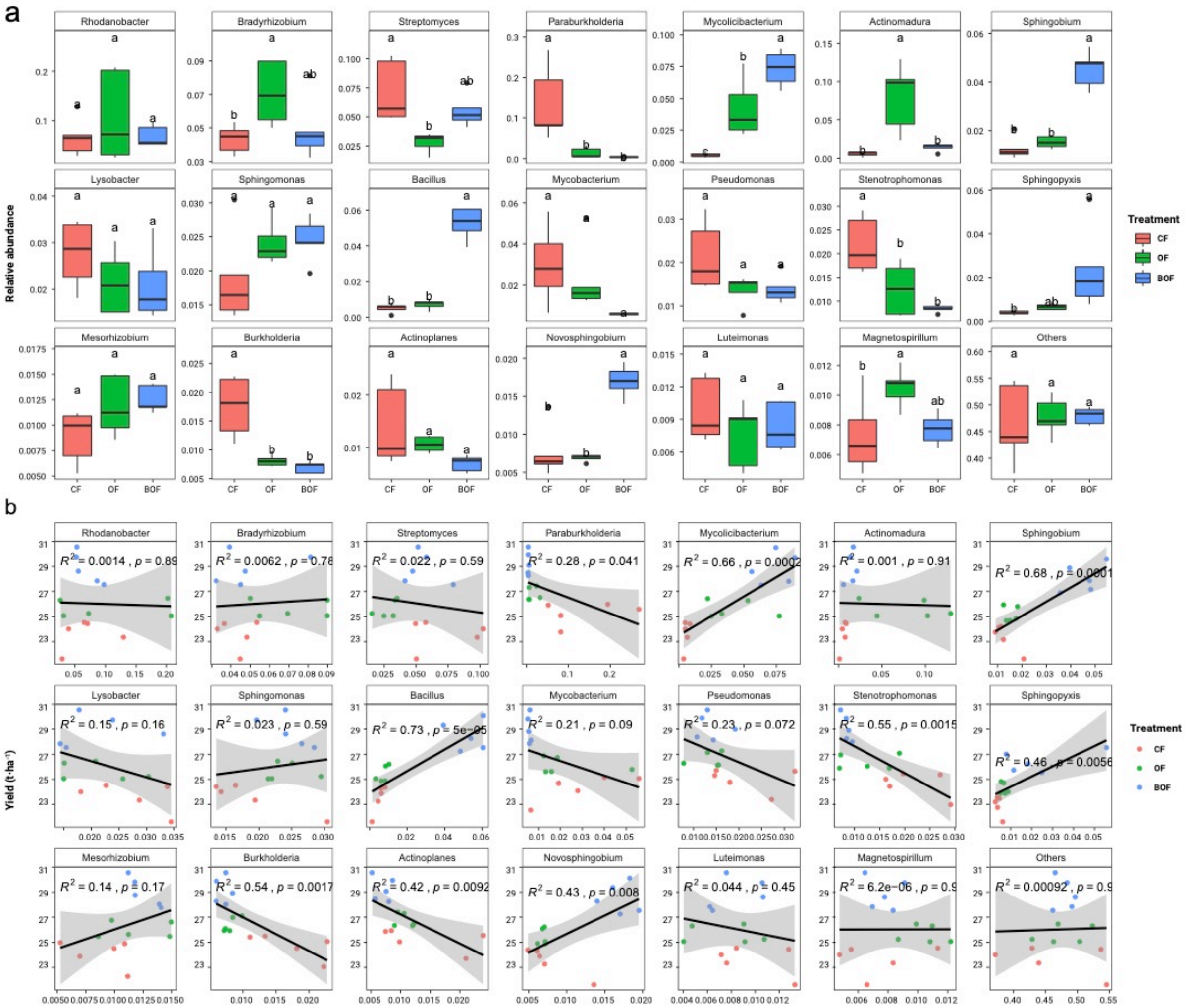

Supplementary Figure 2. Differentially abundant bacterial genera and their associations with pear yield under different fertilization schemes. Panel (**a**) indicates differentially abundant genera in rhizosphere microbiomes. Different small letters above box plots denote for significant differences between treatment groups (P < 0.05). Panel (**b**) shows linear correlations between pear yield and relative abundance of discriminative genera. Black lines indicate linear fitting of curves, while R^2 and p (P-values) indicates the Spearman correlation coefficient and the significance of each correlation. In (**a**) and (**b**), BOF (blue), OF (green) and CF (red) denote for the treatment of bio-organic, organic and chemical fertilizers.

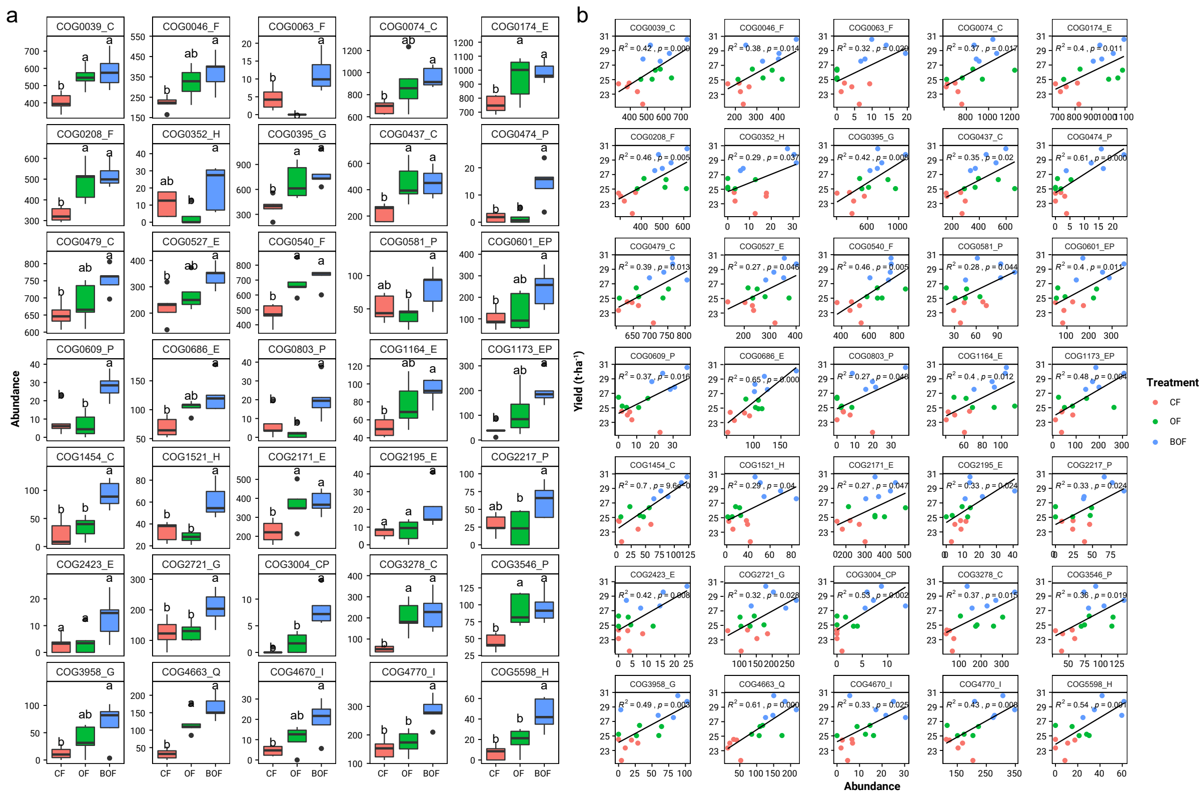

Supplementary Figure 3. Differentially abundant COG functions and their associations with pear yield under different fertilization schemes. Panel (**a**) indicates the differentially abundant COG functions in rhizosphere microbiomes. Different small letters above box plots denote for significant differences between treatment groups (*P* < 0.05). Panel (**b**) shows linear correlations between the pear yield and relative abundance of the discriminative COG functions. Black lines indicate linear fitting of curves, while R^2 and p (*P*-values) indicates the Spearman correlation coefficient and the significance of each correlation. Letters for each title of the facet indicate the COG functions belonging to the categories as following: J: Translation, ribosomal structure and biogenesis; C: Energy production and conversion; E: Amino acid transport and metabolism; O: Posttranslational modification, protein turnover, chaperones K: Transcription; I: Lipid transport and metabolism; F: Nucleotide transport and metabolism; L: Replication, recombination and repair; H: Coenzyme transport and metabolism; R: General function prediction only. In (**a**) and (**b**), BOF (blue), OF (green) and CF (red) denote for the treatment of bio-organic, organic and control fertilizers.

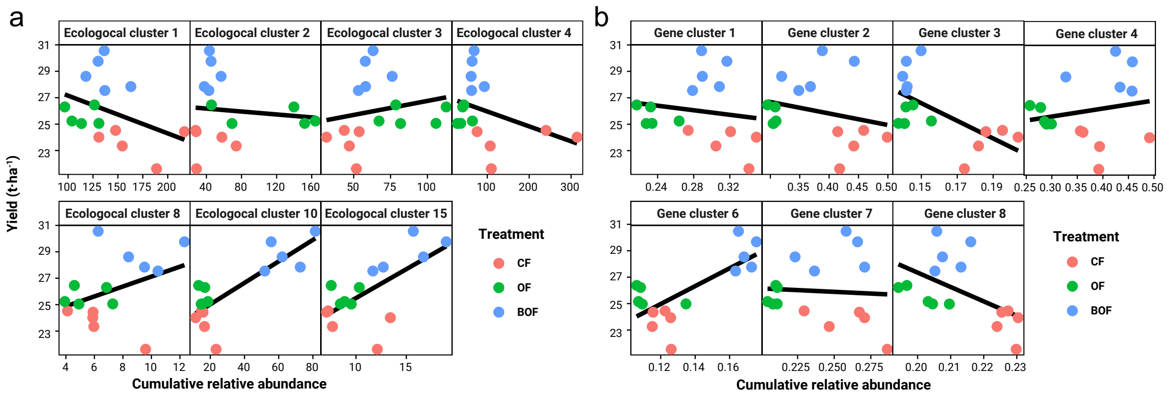

Supplementary Figure 4. Linear correlations between the pear yield and cumulative relative abundance of the ecological (a) and functional (b) clusters. Black lines indices the linear fitting of curves, while R^2 and p (*P*-values) indicates the Spearman correlation coefficient and the significance of each correlation. BOF (blue), OF (green) and CF (red) denote for the treatment of bio-organic fertilizer, organic fertilizer and control fertilizer. M: co-occurrence network module.

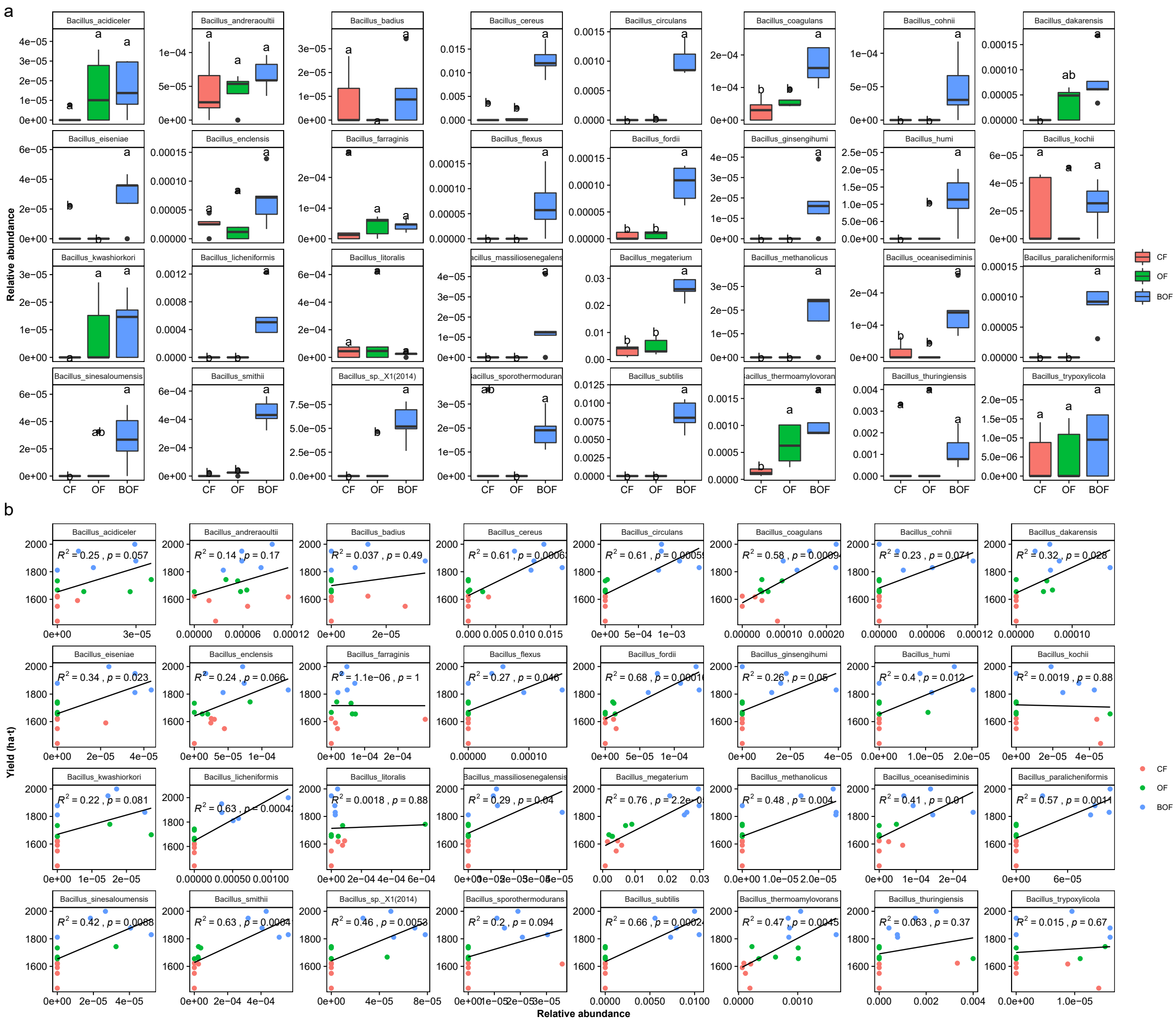

Supplementary Figure 5. Differentially abundant *Bacillus spp*. and their associations with pear yield under different fertilization schemes. Panel (**a**) indicates the differentially abundant *Bacillus spp*. in rhizosphere microbiomes. Different small letters above box plots denote for significant differences between treatment groups (P < 0.05). Panel (b) shows linear correlations between the pear yield and relative abundance of the *Bacillus spp*. In (**a**) and (**b**), BOF (blue), OF (green) and CF (red) denote for the treatment of bio-organic, organic and control fertilizers.
